# Close kinship within multiple-genotype malaria parasite infections

**DOI:** 10.1098/rspb.2012.0113

**Published:** 2012-03-07

**Authors:** Standwell C. Nkhoma, Shalini Nair, Ian H. Cheeseman, Cherise Rohr-Allegrini, Sittaporn Singlam, François Nosten, Tim J. C. Anderson

**Affiliations:** 1 Department of Genetics, Texas Biomedical Research Institute, 7620 NW Loop 410, San Antonio, TX 78227, USA; 2 Malawi-Liverpool Wellcome Trust Clinical Research Programme, University of Malawi College of Medicine, Blantyre, Malawi; 3 Texas Department of State Health Services, San Antonio, TX, USA; 4 Shoklo Malaria Research Unit (SMRU), Maesot, Thailand; 5 Mahidol-Oxford Tropical Medicine Research Unit (MORU), Mahidol University, Bangkok, Thailand

**Keywords:** *Plasmodium falciparum*, multiple-clone infection, dilution cloning, inbreeding, relatedness

## Abstract

Malaria infections containing multiple parasite genotypes are ubiquitous in nature, and play a central role in models of recombination, intra-host dynamics, virulence, sex ratio, immunity and drug resistance evolution in *Plasmodium*. While these multiple infections (MIs) are often assumed to result from superinfection (bites from multiple infected mosquitoes), we know remarkably little about their composition or generation. We isolated 336 parasite clones from eight patients from Malawi (high transmission) and six from Thailand (low transmission) by dilution cloning. These were genotyped using 384 single-nucleotide polymorphisms, revealing 22 independent haplotypes in Malawi (2–6 per MI) and 15 in Thailand (2–5 per MI). Surprisingly, all six patients from Thailand and six of eight from Malawi contained related haplotypes, and haplotypes were more similar within- than between-infections. These results argue against a simple superinfection model. Instead, the observed kinship patterns may be explained by inoculation of multiple related haploid sporozoites from single mosquito bites, by immune suppression of parasite subpopulations within infections, and serial transmission of related parasites between people. That relatedness is maintained in endemic areas in the face of repeated bites from infected mosquitoes has profound implications for understanding malaria transmission, immunity and intra-host dynamics of co-infecting parasite genotypes.

## 1. Introduction

People living in malaria-endemic regions are often infected with *Plasmodium falciparum* infections containing multiple parasite haplotypes [1,2]. These ‘multiple infections’ (MIs) are common, constituting over 70 per cent of human infections in high malaria transmission regions such as sub-Saharan Africa [3,4]. Similarly, MIs are commonly reported in studies of malaria in lizards and non-human primates [5,6]. Although MIs play a prominent role in the models of parasite virulence evolution [7], drug resistance [8,9], sex ratio [10], transmission [11] and population genetics [1], we have minimal understanding of the composition of MIs or the manner in which they are generated.

**Figure 1.**
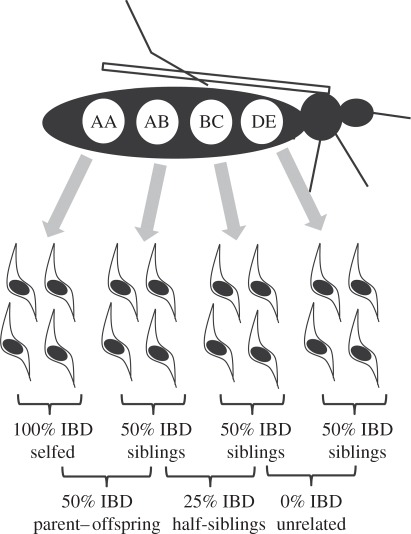
Relatedness among parasites innoculated from a single mosquito. The mosquito has ingested sexual stages of five parasite haplotypes (labelled A–E), which fuse to form diploid zygotes in a variety of combinations. The haploid sporozoites generated by meiosis from each zygote are shown below. The average percentage of the parasite genome that is identity-by-descent (IBD) in pairwise comparisons between sporozoites is shown, together with equivalent relationship categories for diploid organisms.

Malaria parasites are obligately sexual hermaphrodite protists with a predominantly haploid life cycle. In the human host, haploid parasites replicate clonally and differentiate into haploid sexual stages (gametocytes) that are ingested by mosquitoes. Male and female gametes fuse to form a short-lived diploid zygote that divides by meiosis to generate recombinant haploid infective stages (sporozoites). The haploid blood stages are essentially equivalent to gametes of classical diploid organisms. MIs may be generated by successive mosquito inoculation of genetically distinct parasites into the human host (superinfection). This is the usual assumption made in models of malaria transmission, drug resistance and sex ratio [9,10,12], and is based on the observation that relatively few sporozoites (16–64) are inoculated in each infective bite, that further attrition occurs during liver stage infection, and most infective bites do not give rise to infections [13,14]. Under this model, component haplotypes of MIs consist of unrelated parasites randomly sampled from the local population. Alternatively, genetic recombination within the mosquito, which results in the reshuffling of genes and generation of novel recombinant haplotypes, can generate MIs [15]. Under this model, multiple sporozoites from a single mosquito bite establish infections, which will result in related parasite haplotypes within MIs (figure 1). In this case, the number of different haplotypes is limited by the number of oocysts within the mosquito midgut, because each oocyst contains sporozoites derived from the four meiotic products. In reality, these models represent extremes. Superinfection is required to initiate MIs and the fact that it is more common in regions of high transmission is consistent with this [1]. However, the relative importance of superinfection and single mosquito inoculation in generating MIs is unclear.

Genotyping of malaria parasite infections using polymorphic marker loci such as microsatellites or antigenic loci often reveals more than two alleles at individual loci, providing a minimal estimate of the number of haplotypes. However, such data cannot resolve the actual number of haplotypes present. For example, when each of 10 genotyped loci shows two alleles, the total number of haplotypes could range from 2 to 2^10^. Similarly, genetic marker data do not provide information about relatedness between the component haplotypes of MIs because haplotypes cannot be constructed. Deep sequencing of MIs efficiently identifies low frequency alleles on a genome-wide scale and can be used to assess allelic diversity at individual loci [16]. However, similar limitations apply and such data cannot resolve parasite haplotypes present in complex mixtures.

Several studies have attempted to examine the relatedness of parasites within MIs, but all have limitations. Druilhe *et al.* [17] isolated 34 clones from one African patient and 33 clones from one Thai patient and characterized these using antigen, restriction fragment length polymorphism and karyotype markers. They conclude that infections are composed of ‘genetically diverse but related parasites’. However, no quantitative analysis was presented and sampling of a single parasite infection precluded rigorous examination of this question. Conway *et al.* examined MIs from the Gambia [18]. They observed that parasite haplotypes within infections tended to share the same alleles at antigen loci and concluded that infections were frequently founded by single mosquito innoculation of related sporozoites. Similarly, Sutton *et al.* [19] suggested that non-random co-occurrence of multiple allelic types at the merozoite surface antigen 1 locus within MIs in Peru could not be explained by superinfection. However, in both of these studies, the marker loci used are antigens that elicit strong immunity. There is a concern that the patterns observed may therefore reflect immune selection at the loci examined rather than genome-wide patterns of relatedness. Mzilahowa *et al.* [20] genotyped diploid oocysts dissected from mosquitoes. The data from some, but not all loci examined suggested that related parasites are found within hosts. Hence, they too were unable to unambiguously answer this question.

This study was designed to examine the relative contribution of both superinfection and single mosquito bites in generating MIs in Malawi and Thailand, by examination of genome-wide patterns of relatedness among parasite haplotypes within MIs. Malaria transmission is intense in Malawi, with entomological inoculation rates (EIR) as high as 180 infective bites per year [20] and mean malaria prevalence rates of more than 25 per cent in children less than 10 years old [21], and MI carriage rates more than 75 per cent. We therefore predicted that superinfection would be the key generator of MIs in Malawi. On the Thai–Burma border, transmission levels are lower, with an estimated EIR of 0.5–1.5 and *P. falciparum* prevalence rates of 1–4% [22], and MI carriage rates approximately 30 per cent. In this situation, we envisaged that both mechanisms could play a role in generating MIs. To test these predictions, we isolated 336 clones from 14 MIs from Malawi and Thailand, genotyped these using 384 single-nucleotide polymorphisms (SNPs) and used patterns of allele-sharing to assess relatedness.

## 2. Material and methods

### (a) Sample collection

In Malawi, we collected venous blood samples from malaria patients less than 5 years old with confirmed *P. falciparum* malaria and a haematocrit of more than or equal to 20 per cent presenting to Ndirande Health Centre, Blantyre (April–June 2008). Informed consent was obtained from parents or guardians and the study was approved by the College of Medicine Research and Ethics Committee, University of Malawi. In Thailand, 5 ml blood samples were collected from malaria patients presenting to the Mawker-Thai clinic on the Thai–Burma border (March–October 2003).

### (b) Identification of multiple infections

Malawi: we genotyped 79 infections using seven microsatellite markers to identify MIs [23]. MIs were defined as those in which one or more of the seven loci showed multiple alleles. Sixty-two out of 79 Malawi infections (78%) were MIs. Eight randomly selected MIs were cloned by limiting dilution.

Thailand: we identified MIs by examining length variation in glutamate-rich protein, merozoite surface protein-1 and merozoite surface protein-2 [24] within 24 h of sample collection. Infections were defined as MIs if more than one variant was present at one or more of these loci. Six randomly selected MIs were cloned by limiting dilution.

### (c) Dilution cloning and detection of positive wells

Procedures used in Malawi and Thailand differ in several respects.

#### (i) Malawi

Parasite isolates were adapted to *in vitro* culture for approximately three weeks before cloning. To maximize the probability of isolating parasite lines derived from single cells, we avoided cloning from cultures with erythrocytes infected with more than one parasite and parasitaemias greater than 2 per cent. We innoculated 250 µl of parasite culture containing approximately one parasite per millilitre into each well of a 96-well plate. The media was changed daily for 7 days and every 2 days for the next 14 days. On day 20, positive wells were identified by detection of parasite DNA using Sybr green 1 and cloning was discontinued if plates yielded the expected number of clones (assuming a Poisson distribution, parasites are expected in approx. 21 wells, with 19 seeded with a single parasite). Otherwise, plates were incubated for 10 more days. Parasite positive wells were confirmed by microscopy and expanded to obtain sufficient material for DNA and cryopreservation.

#### (ii) Thailand

Parasite isolates were cloned by limiting dilution as described above but without culture adaptation. Beginning on day 22 of cloning and continuing every third day until day 60, we identified parasite positive wells using the Malstat LDH assay [25]. Positive wells were expanded to obtain sufficient material for cryopreservation and DNA.

### (d) Single-nucleotide polymorphism genotyping

We used the Illumina GoldenGate platform to genotype 384 SNPs distributed across all 14 chromosomes of *P. falciparum* genome (electronic supplementary material, figure S1 and table S1). The SNPs were chosen using the Plasmodb v. 6.2 (www.plasmodb.org) to maximize variability in African samples (electronic supplementary material, table S1). SNP genotyping was carried out according to the Illumina instructions except we used 100 ng DNA template. We included DNA from parasites 3D7, HB3, FCR3-FMG, FCB, K1, D6, R033, W2 and DD2 as controls.

### (e) Allele frequency estimation

To investigate the efficiency of cloning, we compared the allele frequencies of SNPs within uncloned MIs from patients with that observed in the component clones isolated from each MI. In the case of uncloned MIs, allele frequencies were estimated for each SNP from the GoldenGate readout (electronic supplementary material, figure S1). SNPs that are polymorphic within MIs fall between the clusters formed by haploid parasites bearing each of the alternative bases. The position of these ‘heterozygous’ SNP calls provides a crude estimate of allele frequency and is recorded by the *θ*-value (see the electronic supplementary material, figure S1 for estimation). For cloned progeny, we counted alleles present in all clones isolated from MI to estimate allele frequencies. The correlation between-infection-based and clone-based allele frequencies for each SNP was assessed using Spearman rank correlation in the R statistical environment (http://www.Rproject.org).

### (f) Parasite relatedness structure

We calculated the proportion of alleles shared (*ps*) between haplotypes recovered from the same infection and those from different infections at each location. We constructed unweighted pair group method with arithmetic mean (UPGMA) trees based on the metric 1*-ps* using PhylIP (http://evolution.genetics.washington.edu/phylip.html) to examine patterns of relatedness between parasite haplotypes, and computed mean *ps* for both within- and between-infection haplotype comparisons. We then permuted haplotypes among hosts 10 000 times and recalculated the difference between these statistics each time to determine whether the observed difference in relatedness could be generated by chance.

To provide a biological interpretation of the relatedness between parasite haplotypes within infections, we simulated allele-sharing expected for unrelated parasites, and for haploid parasites derived from the same zygote (siblings), and parasites sharing one zygotic parent (half-siblings) [26]. These simulations were conducted using observed allele frequencies for each SNP derived from single-haplotype parasite isolates sampled from the same clinics in both Thailand and Malawi. Explanations of relatedness categories and terminology is provided in figure 1.

## 3. Results

### (a) Cloning and single-nucleotide polymorphism genotyping of multiple infections

**Figure 2.**
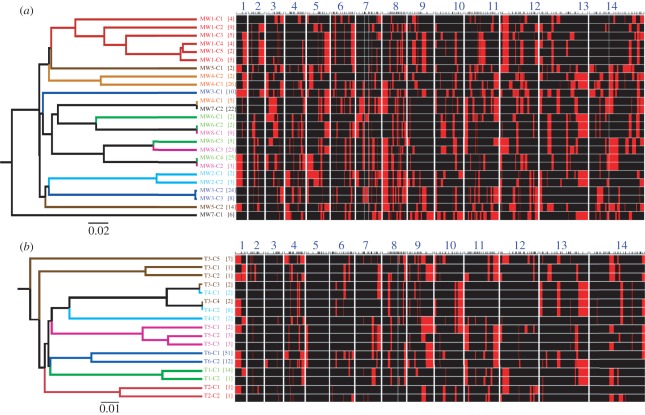
Haplotype structure of parasites within MIs. Haplotypes recovered from MIs in (*a*) Malawi and (*b*) Thailand. Chromosome blocks marked by major and minor SNP alleles are shown in black and red, respectively (right panel), with numbers 1–14 representing the 14 *P. falciparum* chromosomes. The locations of genotyped SNPs are shown by tick marks above each chromosome. UPGMA tree (left panel) shows clustering of haplotypes based on allele-sharing. Patient codes (MW1–8 from Malawi and T1–6 from Thailand) are shown on the right of the tree, while the tree branches and labels are coloured to reflect the patient from which each parasite haplotype was recovered. Square brackets contain the number of clones isolated for each haplotype.

We isolated 114 individual clones from 6 MIs in Thailand and 222 clones from eight Malawi MIs. In addition, we identified 71 single-haplotype infections from Thailand and 17 from Malawi to assess population allele frequencies. We genotyped these using 384 SNPs distributed across the *P. falciparum* genome (electronic supplementary material, table S1). We excluded 19 SNPs, which could not be easily scored, and 49 SNPs with more than 5 per cent missing data, leaving 316 SNPs. Of these, 270 (85%) were variable in Malawi while 145 (46%) in Thailand. The 114 Thai clones clustered into 15 distinct haplotypes, each represented by 1–51 clones, with two to five distinct haplotypes per infection. In Malawi, 22 distinct haplotypes were found in eight MIs, with two to six distinct haplotypes per infection and 2–26 clones isolated per haplotype (figure 2 and table 1).

**Table 1.** Comparison of parasite relatedness in Thailand and Malawi. ((*a*) Simulated data. Upper and lower 95% CI for expected allele-sharing for different categories of parasite relatedness generated through simulations (figure 3*c*,*d*). These simulations provide conservative thresholds for categorizing unrelated, related and extremely related parasites. The upper CI for simulations of unrelated parasites provides the threshold for distinguishing unrelated and related parasites, while the upper CI for simulations of full-sibling parasites provides the threshold for distinguishing related and extremely related parasites. (*b*) Observed data. Summary of haplotype sampling and patterns of relatedness, within and between MIs. Parasites were categorized as related if they showed greater allele-sharing than expected for unrelated parasites. Parasites showing *extreme* relatedness were defined as those with greater allele-sharing than that expected for ‘full-sibling’ parasites derived from the same oocyst.)

	Malawi	Thailand
(*a*) simulated data		
unrelated (0% IBD)	0.646–*0.741*^a^	0.784–*0.858*^a^
half-sibling (25% IBD)	0.724–0.813	0.829–0.899
full-sibling (50% IBD)	0.807–*0.883*^b^	0.880–*0.940*^b^
1 generation inbreeding	0.892–0.953	0.934–0.975
2 generation inbreeding	0.940–0.981	0.962–0.993
AA (fully inbred)	100	100
(*b*) observed data		
MIs sampled	8	6
clones isolated	222	114
haplotypes found	25	17
haplotypes per infection	2–6	2–5
infections containing related parasites	6/8	6/6
% related *within* host comparisons (*n*)^c^	66.7 (33)	47.4 (19)
% related *between* host comparisons (*n*)^c^	1.5 (267)	3.4 (117)
probability relatedness within infections^d^	0.54	0.65
% *extreme* relatedness within infections	15.2	21.1
% *extreme* relatedness between infections	1.5	3.4

^a^Thresholds for defining relatedness.

^b^Thresholds for defining extreme relatedness.

^c^*n* is the number of pairwise comparisons.

^d^Mean values were weighted by numbers of haplotypes recovered from each MI.

### (b) Genetic composition of multiple infections

**Figure 3.**
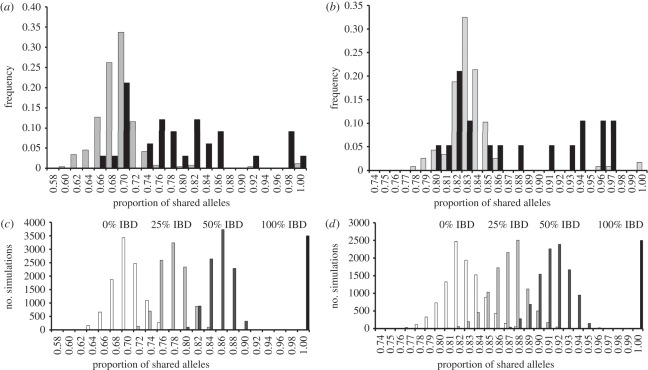
Relatedness structure of parasites within MIs. (*a*,*b*) Observed frequency distribution of pairwise allele-sharing (*ps*) between haplotypes recovered from the same or from different MIs in Malawi and Thailand. Grey bars denote between infections and black bars denote within infections. (*c,d*) Simulated *ps* distributions for parasites in different relatedness classes in Malawi and Thailand. We simulated *ps* expected in parasites derived from the same inbred oocyst (100% IBD), from the same outcrossed oocysts (50% IBD), from two related oocysts (25% IBD) and from two unrelated oocysts (0% IBD). Upper and lower CI for these distributions are shown in table 1.

Component haplotypes of MIs share identity on many chromosomes (figure 2). For example, patient T5 harbours three parasite haplotypes that are identical on chromosome 4, which are not observed in the other Thai MIs. Similarly, four parasite haplotypes from patient MW1 contain an identical chromosome 5 region that is not observed in other parasites from Malawi. UPGMA trees show that in both Malawi and Thailand, parasites from the same MI tend to cluster together (figure 2) suggesting that they are more closely related to each other than to parasites from other infections. The frequency distribution of pairwise relatedness between haplotypes confirms this (figure 3*a,b*). In both countries, pairwise relatedness among parasites is greater within than between MIs. In Malawi, parasite haplotypes shared on average 78.7 per cent of alleles (range: 65.2–99.7%) within MIs compared with 68.3 per cent (range: 59.2–100%) in comparisons between MIs. Equivalent figures for Thailand were 87.6 per cent (range: 79.7–96.2%) within infections and 82.9 per cent (range: 77.8–100%) between infections. Permutation analyses showed that haplotypes within MIs are significantly more related than expected by chance in both Thailand (*p* = 0.0001) and Malawi (*p* < 0.0001).

We observed the same haplotypes occurring in independent MIs at both locations. In Thailand, one identical haplotype was found in two different MIs (T3-C4/T4-C2), while two nearly identical haplotypes differing by just 1/145 SNPs were found within the same two infections (T3-C3/T4-C1) (figure 2). Similarly, in Malawi, two patients (MW6 and MW8) carried identical pairs of haplotypes (MW6-C2/MW8-C1 and MW6-C4/MW8-C2), in addition to two haplotypes (MW6-C3/MW8-C3) that differed at just 28/316 alleles.

### (c) Relatedness within multiple infections

Are parasites within MIs more or less related than first-order relatives? To address this question, we simulated allele-sharing expected among unrelated parasites, among progeny haplotypes from the same oocyst and among haplotypes derived from inbreeding among progeny (figure 3*c*,*d*). These simulations were conducted using observed allele frequencies derived from single-haplotype infections sampled from the same clinics in both Thailand (*n* = 71) and Malawi (*n* = 17) and provide threshold levels of allele-sharing for grouping pairwise comparisons of parasite haplotypes into the different relatedness classes (table 1). As the allele-sharing distributions for different relatedness categories show some overlap, we used conservative cut-offs to differentiate between related and unrelated haplotypes pairs (greater than 95% of simulated values for unrelated haplotypes). In both locations, most comparisons between haplotypes from different MIs fall into the relatedness class expected for unrelated parasites, with the exception of a small number of haplotypes that are identical or closely related (2.6 and 3.4% of comparisons in Thailand and Malawi, respectively; table 1). Relatedness within MIs is dramatically different: 66 per cent and 47 per cent of all pairwise haplotype comparisons are related at the half-sibling level or greater in Malawi and Thailand, respectively. These estimates may be biased because unequal numbers of haplotypes are recovered from different patients. Following weighting of this estimate by the number of haplotypes within each patient, the mean probability of picking two related haplotypes from MIs was 0.65 in Thailand and 0.54 in Malawi.

### (d) Extreme relatedness within infections

**Figure 4.**
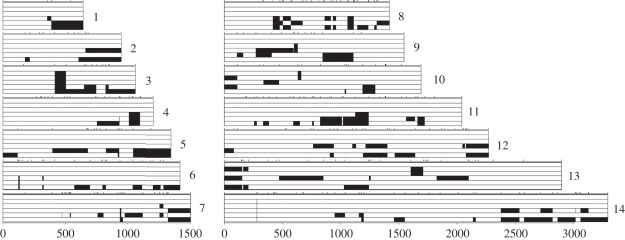
Extreme relatedness within MIs. Haplotypes recovered from patient MW1 in Malawi. All 14 chromosomes are shown and the six bars denote the six haplotypes recovered (MW1-C4, C5, C6, C3, C2, C1 from top to bottom). SNP calls from the reference haplotype (MW1-C4) are coded in white, while blocks defined by divergent SNPs are shown in black. Haplotypes MW1-C4 and MW1-C5 are indistinguishable on 11/14 chromosomes and differ by nine SNPs in four chromosome blocks. The locations of SNPs genotyped are shown by tick marks above each chromosome.

Some haplotypes within MIs show extreme relatedness. These parasites share more alleles than would be expected for full-siblings, but are not identical. These constitute 15.2 per cent of parasites within Malawi MIs and 21.1 per cent of parasites within Thai MIs. The haplotypes differ at less than 32 SNPs in Malawi and less than nine SNPs in Thailand. For example, in the infection MW1 from Malawi, haplotypes MW1-C4 and MW1-C5 only differ at 9/316 SNPs (figure 4), while MW3-C2 and MW3-C3 in patient MW3 differ by just one SNP on chromosome six (electronic supplementary material, table S2).

Three observations suggest that the SNP differences observed in closely related haplotypes cannot be explained by genotyping error. First, the SNPs observed were recorded in multiple independent clones isolated from each MI. For example, the 1 bp difference between MW3-C2 and MW3-C3 is determined from each of the 24 and eight identical clones representing these haplotypes. Second, divergent SNPs in closely related parasites occured in blocks along each chromosome (figure 4). For example, the nine SNP differences between MW1-C4 and MW1-C5 are clustered on chromosomes seven, nine, 10 and 13. Third, divergent blocks are identical in sequence to those from other parasites within the same MI, consistent with generation of differences through recombination. For example, the divergent region of chromosome 13 in MW1-C5 is identical to one in MW1-C1, but is not seen in any other parasites from Malawi (figure 4).

### (e) Comparison of pre- and post-cloning parasite populations

**Figure 5.**
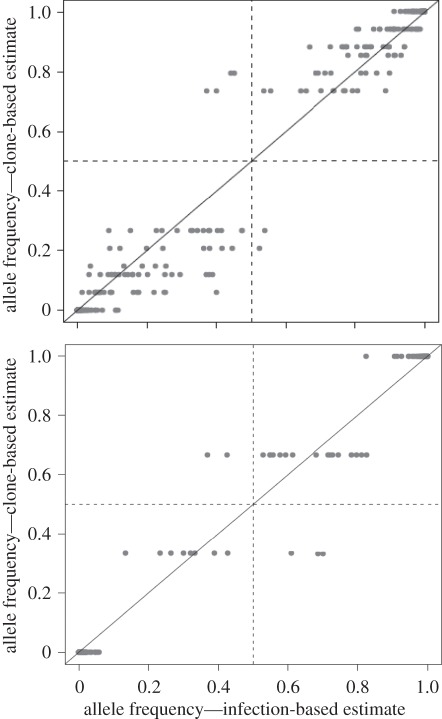
Assessment of cloning bias. Plots show the relationship between SNP allele frequencies of MIs estimated from uncloned parasites within patient blood samples (*x*-axis) and those estimated from parasite clones isolated from each MI (*y*-axis). Spearman rank correlation *ρ*-values were 0.97 for patient MW6 and 0.91 for patient T2. *ρ-*values for the remaining 12 MIs ranged from 0.69 to 0.96 (electronic supplementary material, figure S2).

We were concerned that selection of related sub-populations of haplotypes during cloning could generate the observed relatedness patterns. We therefore compared the allele frequencies of SNPs within blood samples from patients with that observed in the parasite clones isolated from these patients. In general, we found good correspondence between allele frequencies at the 316 SNPs estimated before and after cloning (Spearman rank correlations, *ρ* = 0.69–0.97, *p* < 0.001 for all infections; figure 5; electronic supplementary material, figure S2). These comparisons suggest that the haplotypes isolated correspond well to those present within MIs.

## 4. Discussion

### (a) Parasite relatedness within multiple infections

Models of malaria transmission generally assume that only one sporozoite from a single mosquito bite establishes a blood-stage infection [12] and that MIs are comprised of unrelated parasites randomly sampled from the parasite population. However, our data reveal that parasite haplotypes recovered from within MIs tend to be more related than haplotypes isolated from different MIs. We quantified patterns of relatedness using simulations to estimate expected allele-sharing among unrelated, half-sibling and full-sibling parasites. Using threshold relatedness estimates (table 1) derived from these simulations, we inferred that all six Thai MIs (100%) and six of eight Malawi MIs (75%) contained haplotypes that were consistent with sibling or half-sibling relationships (figures 1 and 3). Approximately, half of the pairwise comparisons of haplotypes within infections (66.7% in Malawi, 47.4% in Thailand) were consistent with relatedness at the half-sibling level or greater. We emphasize that these simulation-based thresholds underestimate the proportion of related parasites, so are likely to be conservative. The most plausible explanation for these results are that: (i) single mosquito inoculation of related parasites generated by meiosis in the mosquito midgut constitute a large proportion of parasites within MIs, and (ii) blood-stage infections are frequently founded by more than one infective sporozoite. While we do see unrelated parasites (table 1) within infections, related parasites from single mosquito bites contribute more significantly to in-host parasite diversity than superinfection in both Malawi and Thailand.

### (b) Maintenance of parasite relatedness in spite of repeated mosquito inoculation

Our results are consistent with low levels of malaria transmission in Thailand [22], where most people receive less than one infective bite per year. However, they are at odds with the high levels of malaria transmission in Malawi [20,21], where people receive multiple infective bites per night and would be expected to contain multiple unrelated haplotypes. How are the observed patterns of relatedness maintained in the face of high levels of malaria transmission? We suggest several possible explanations.

#### (i) Immunological explanation

The patterns observed may reflect intra-host dynamics driven by immunity. Despite repeated inoculation of new parasites, only related parasites that share novel alleles at immunogenic loci may reach high frequencies within infections. The *var* gene family underlies antigenic variation in malaria, plays a key role in intra-host dynamics and shows highly structured, genotype-specific switching patterns [27], consistent with this reasoning. Two lines of empirical evidence suggest that the duration of infection of newly inoculated parasites may be limited in endemic areas. In longitudinal studies of asymptomatic infections, parasites bearing particular marker loci remain detectable within infections for a short window of time before being replaced by a different set of parasites [28–30]. Similarly, symptomatic infections have been associated with uncontrolled growth of new haplotypes that escape immune control [31,32]. Under this model, peripheral blood may contain only recently inoculated families of parasites, generating the observed relatedness structure.

#### (ii) Supression of superinfection

Recent work on the rodent malaria model shows that above a certain critical threshold of parasite density, an ongoing blood-stage infection inhibits the growth of subsequently inoculated sporozoites in liver hepatocytes, preventing them from becoming blood-stage parasites [33]. If this phenomenon occurs in human infections, it would effectively limit the levels of superinfection and contribute to patterns of relatedness observed. Similarly, acquired malaria immunity (premunition) could suppress superinfection [34], generating patterns observed. However, we do not think this is a major factor because we studied MIs from children with little or no premunition.

#### (iii) Localized variation in allele frequencies

Localized variation in allele frequencies provides an alternative explanation for the patterns observed. If mosquitoes within a local area tend to carry related parasites, then superinfection could generate similar patterns to those observed. We think this is unlikely to explain these results, because analyses of parasite genetic structure at the level of individual mosquitoes, households and villages reveal no such evidence for microgeographical structure of parasite populations [35].

### (c) Extreme relatedness among haplotypes

We found that 15.2 per cent of parasites within Malawi MIs and 21.1 per cent of parasites within Thai MIs show levels of allele-sharing that exceed those expected for siblings (i.e. meiotic products from the fusion of two unrelated parental parasites). We suggest three processes that may be responsible for generating such closely related haplotypes. First, if MIs are serially transmitted from one patient to another as intact units, then repeated inbreeding will progressively diminish genetic variation among constituent haplotypes as occurs during the generation of recombinant inbred lines in laboratory model organisms [36]. Second, these high levels of relatedness could be generated by biased inheritance of chromosomal segments from one parental genome during meiosis. This process results in over-representation of one of the two parental genomes among the progeny and is observed in laboratory crosses of *P. falciparum* [37,38]. Finally, extreme levels of relatedness could be generated by mitotic recombination among co-infecting parasite haplotypes. Ectopic recombination has been reported in telomeric regions in *P. falciparum* during asexual propagation [39], although mitotic recombination events are not currently thought to occur between different malaria haplotypes within the same MI. Our data provide some support for the serial transmission model, because we found that identical or closely related haplotypes are shared between MIs in both Malawi and Thailand. Identical parasite haplotypes are often recovered from different patients in low-transmission regions such as southeast Asia and South America, but are rarely observed in African countries. We suggest that these may be common in high transmission regions but are rarely observed because MIs predominate and obscure identification of such haplotypes.

### (d) Implications for malaria biology and control

#### (i) Models of malaria transmission and drug resistance

Mathematical models of malaria transmission and drug resistance assume that only a single sporozoite is the founder for blood-stage infections from a mosquito inoculation [9,12]. Similarly, methods for maximum-likelihood estimation of allele frequencies and numbers of haplotypes per infection are based on the assumption that component haplotypes within infections are independently sampled from the parasite population [12,40,41]. This assumption is violated if the haplotypes within infections are related and therefore not independent [40]. Relatedness among haplotypes within MIs influences multiple aspects of resistance evolution, by reducing the importance of competitive release [42], and the rate at which multi-locus combinations are broken apart by recombination [9]. Refined models will be required to evaluate the impact of relatedness structure on resistance evolution.

#### (ii) Evolution of parasite virulence and sex ratio

Kin selection plays a central role in models of intra-host interactions [43] and has been widely applied to understand virulence and sex ratio in malaria parasites [7,10,44]. Competition between haplotypes within MIs is predicted to result in increased virulence, because haplotypes which grow most aggressively within hosts will maximize both damage to the host and transmission. Similarly, relatedness among co-infecting haplotypes is expected to favour female-biased sex ratios and reduced virulence. Rodent model malaria systems are extensively used to experimentally test models of virulence, sex ratio and competition in malaria infections [45,46], but mixed infections are generally constructed using unrelated parasite haplotypes. Empirical data from human *P. falciparum* malaria do not always show a consistent positive relationship between parasite virulence and multiple-haplotype infections [47]. Conflicting conclusions from experimental work on rodent malaria and human studies could be reconciled if most MIs in human infections consist of related parasites.

#### (iii) Inbreeding in malaria

Inbreeding levels determine rates of effective recombination and play a central role in understanding the population genetics of malaria. Genotyping of oocysts from the mosquito midgut, the stage containing the haploid products of meiosis, has been used to directly measure heterozygote deficit and inbreeding rates in malaria. These estimates range from 0.30 to 0.90, and are surprisingly high even in regions where MIs predominate [48]. The close relatedness observed within MIs is expected to reduce levels of outcrossing in malaria and provides an additional component of inbreeding in malaria.

#### (iv) Genetic mapping

Linkage mapping has been extremely successful for mapping biomedically important malaria traits, but crosses are expensive, labour intensive and ethically problematic [49]. Our results show that the progeny of natural genetic crosses can be sampled from natural infections. We envisage that isolation and phenotyping of arrays of related haplotypes from MIs will allow novel mapping strategies that combine benefits of both linkage (large blocks of chromosomal similarity) and association mapping (efficient sampling of phenotypic variation in nature). We note that sampling of progeny arrays has proved a powerful approach for genetic mapping in mosquito vectors [50].

#### (v) Detection of multiple infections

Related parasites complicate detection of MIs. A few microsatellite or antigen markers currently used to identify MIs may not accurately distinguish MIs from single-haplotype infections when closely related parasites are present. Two progeny from a genetic cross are expected to be identical-by-descent at 50 per cent of the genome. Hence, use of a single marker will have a 0.5 probability of detecting an MI with this composition, while three polymorphic markers will have a (0.5^3^ = 0.125) of mis-scoring the infection as single-haplotype.

In conclusion, genetic dissection of small numbers of MIs from two locations reveal surprising patterns of relatedness among component parasite haplotypes and suggest that simple superinfection models are inadequate to explain the genetic complexity observed within *Plasmodium* infections. These results refine our understanding of *P. falciparum* transmission dynamics and population structure and have important implications for malaria control, but raise many further questions about intra-host dynamics and structure of malaria infections.
